# The Impact of Vitamin E Supplementation on Oxidative Stress, Cognitive Functions, and Aging‐Related Gene Expression in Aged Mice

**DOI:** 10.1002/fsn3.4548

**Published:** 2024-10-26

**Authors:** Farnoosh Molavi Vasei, Mohammad Yasin Zamanian, Maryam Golmohammadi, Mehdi Mahmoodi, Morteza Khademalhosseini, Tayyebeh Tavakoli, Ozra Sadat Esmaeili, Sadegh Zarei, Mohammad Reza Mirzaei, Mohammad Reza Hajizadeh

**Affiliations:** ^1^ Department of Clinical Biochemistry, School of Medicine Rafsanjan University of Medical Sciences Rafsanjan Iran; ^2^ Neurophysiology Research Center Hamadan University of Medical Sciences Hamadan Iran; ^3^ Department of Pharmacology and Toxicology, School of Pharmacy Hamadan University of Medical Sciences Hamadan Iran; ^4^ School of Medicine Shahid Beheshti University of Medical Sciences Tehran Iran; ^5^ Department of Clinical Biochemistry, Afzalipoor Faculty of Medicine Kerman University of Medical Sciences Kerman Iran; ^6^ Department of Pathology, School of Medicine Rafsanjan University of Medical Sciences Rafsanjan Iran; ^7^ Department of Physiology‐Pharmacology, School of Medicine Rafsanjan University of Medical Sciences Rafsanjan Iran

**Keywords:** aging, mice, oxidative stress, vitamin E

## Abstract

This study investigated the effects of different doses of vitamin E on oxidative stress, cognitive function, and gene expression in aged mice. A total of 32 male mice, aged 12 months, were divided into a control group and three treatment groups. These groups received varying daily doses of vitamin E for a period of 28 days. The results showed significant improvements in cognitive function, specifically in working memory and spatial learning, in the groups that received vitamin E (100, 200, or 400 mg/kg) compared to the control group. The markers of oxidative stress and antioxidant enzyme activities also demonstrated improvements, with higher doses of vitamin E showing greater effects. The analysis of gene expression revealed increased expression of SIRT1, Nrf2, and Calstabin2, particularly at higher doses of vitamin E. These findings suggest that vitamin E supplementation may help counteract age‐related cellular changes. The study concludes that vitamin E supplementation can reduce oxidative stress, enhance cognitive function, and affect genetic markers of aging in mice, which may have therapeutic benefits in addressing age‐related cognitive decline and oxidative damage. Further research is necessary to investigate the clinical implications of these findings in humans.

## Graphical abstract

Vitamin E supplementation enhanced working memory and spatial learning, decreased oxidative stress, and positively affected the expression of key aging‐related genes such as SIRT1, Nrf2, and Calstabin2. The benefits of vitamin E were found to be dose‐dependent, with higher doses yielding more significant improvements. Additionally, vitamin E influenced the activity of antioxidant enzymes like SOD and GPX and reduced levels of MDA, a marker of oxidative stress. The study highlights the potential of vitamin E supplementation in mitigating age‐related changes in the heart and brain, though further research is needed to confirm these findings and explore clinical applications.

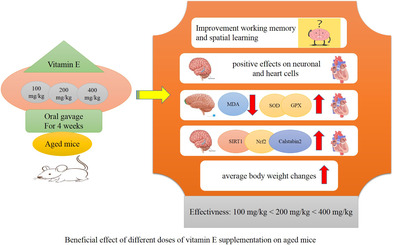

**Abbreviations**
GPX: glutathione peroxidase
MDA: malondialdehyde
Nrf2: nuclear factor‐erythroid factor 2‐related factor 2
ROS: reactive oxygen species
RyR2: ryanodine receptor 2
SIRT1: NAD‐dependent deacetylase sirtuin‐1
SOD: superoxide dismutases

## 1 Introduction

Aging is an inherent biological process that involves the gradual buildup of detrimental changes in cells as we get older. This accumulation of damage to cells results in impaired cellular function and disrupts the balance within tissues, ultimately raising the susceptibility to disease and mortality (da Costa et al. 2016; Tsesmelis et al. 2023). The process of aging increases the likelihood of developing different diseases associated with advancing age, such as heart conditions, neurodegenerative disorders, and metabolic dysfunctions and immune system dysfunction (Chalise 2019; Pizza et al. 2011; Franceschi et al. 2018). As individuals age, there's a greater likelihood of experiencing cognitive impairment, marked by a deterioration in cognitive functions encompassing memory, attention, language, and issue‐resolving capabilities (Kirova, Bays, and Lagalwar 2015). Advancing age significantly increases the susceptibility to neurologic conditions such as Alzheimer's disease (AD), Parkinson's disease (PD), and vascular dementia (Tchekalarova and Tzoneva 2023; Mariani et al. 2005). These diseases involve the progressive degeneration of neuronal cells and may result in significant cognitive decline (Farooqui and Farooqui 2009).

Alterations in cardiac structure and functionality with age and illness correlate with modifications in signaling pathways and gene expression within the heart transcriptome. Such changes transpire naturally over time and are also evident in disease states (Lazzeroni et al. 2022).

Oxidative stress contributes to aging by causing harm to cells, tissues, and organs (Hajam et al. 2022). The accumulation of ROS, arising from an assortment of intrinsic and extrinsic processes, can overpower the antioxidant defenses of the body (He et al. 2017). This disparity in ROS production and antioxidant defense results in oxidative stress (Salisbury and Bronas 2015; Liguori et al. 2018; Miquel 2009).

Endogenous antioxidants (superoxide dismutase, SOD, and glutathione peroxidase, GPX) are naturally produced by the body to counteract the harmful effects of ROS and oxidative stress. These antioxidants perform a vital function in preserving cellular health and preventing damage caused by free radicals (Ighodaro and Akinloye 2018; Yin, Han, and Cao 2019). MDA is a byproduct of lipid peroxidation, which occurs when ROS attack and damage lipids in cell membranes. MDA is recognized as an indicator of oxidative stress, as its levels rise in response to a disproportion between reactive oxygen species (ROS) generation and the body's antioxidative defense mechanisms (Ahsani and Fidianingsih 2018).

Roughly one‐third of the variability in human longevity can be attributed to genetic factors. Over time, changes occur in the levels of aging regulators, causing various aspects of aging. Altering the expression of genes linked to aging may contribute to a prolonged life span and promote a more robust aging process, presenting a potential focus for therapeutic strategies (Murabito, Yuan, and Lunetta 2012). SIRT1, Nrf2, and Calstabin2 are considered as important age‐related genes.

SIRT1, identified as a sirtuin family protein, plays a pivotal role in various cellular mechanisms (Chang and Guarente 2014). As an NAD^+^‐dependent protein deacetylase, SIRT1 is responsible for detaching acetyl groups from proteins (Yu and Auwerx 2010). It is essential in controlling cell functions including metabolism, DNA repair, inflammation, and the aging process (Hwang et al. 2013). Stimulation of SIRT1 has been observed to exert advantageous impacts on conditions and diseases linked to aging (Guo et al. 2022). Studies have demonstrated that SIRT1 activation can safeguard neurons from oxidative stress, inflammatory responses, and neurologic conditions such as AD and PD (Singh, Hanson, and Morris 2017; Gomes et al. 2018). Furthermore, enhanced cognitive abilities and memory in the context of aging have been associated with SIRT1 activation (Cao, Dou, and Li 2018). In cardiac health, SIRT1 activation is known to defend against age‐induced cardiac dysfunction and damage (Alcendor et al. 2007). It helps maintain cardiac function and structure during aging by reducing oxidative stress, inflammation, and apoptosis (Hosoda et al. 2023; Yang et al. 2018).

Nrf2 is an essential transcription factor involved in safeguarding cells against oxidative stres (Zamanian et al. 2023a). It is instrumental in regulating the antioxidant response and maintaining redox balance (Zamanian et al. 2023b). Stimulation of the Nrf2 pathway results in increased synthesis of antioxidative enzymes including SOD, catalase, and GPX. These enzymes are vital in counteracting ROS and reducing oxidative harm in the brain (Zamanian et al. 2023c).

Calstabin2, alternatively recognized as FKBP12.6, constitutes one of the subunits within the RyR2 macromolecular complex (Huang et al. 2006). It is vital in maintaining and controlling the activity of RyR2, a calcium release channel present in cardiac muscle tissue (Wehrens, Lehnart, and Marks 2005). Calstabin2 is pivotal in maintaining RyR2 in its closed state, effectively hindering the leakage of calcium into the cytoplasm (Marks 2013). Dysfunction or downregulation of calstabin2 has been associated with cardiac aging and various cardiovascular diseases (Salemi et al. 2023; Njegic, Wilson, and Cartwright 2020). Furthermore, recent research indicates that calstabin2 might also contribute to brain functionality, particularly in spatial and emotional memory (Salemi et al. 2023; Han et al. 2020). Calstabin2 has been implicated in the regulation of working memory (Salemi et al. 2023). Working memory denotes the cognitive ability to briefly retain and process information within the mind for cognitive tasks (Chai, Abd Hamid, and Abdullah 2018).

Research has indicated that consuming an antioxidant‐rich diet can lower the likelihood of age‐related diseases and support a healthy aging process (Ochi and Takeda 2015; Galli and Azzi 2010; Hashem et al. 2016).

Vitamin E consists of eight similar lipid‐soluble chromanol compounds. It occurs naturally in various food sources and is composed of tocopherols consisting of saturated phytyl tails, while tocotrienols possess unsaturated phytyl tails containing three double bonds (Joshi and Praticò 2012). Vitamin E is a powerful antioxidant that aids in safeguarding cells against oxidative damage caused by free radicals (Singh et al. 2005). It helps reduce oxidative stress and inflammation, which are associated with senescence and age‐associated disorders (Singh et al. 2005; Ulatowski and Manor 2015). Vitamin E supplementation was found to have a positive effect on working memory in older adults (Power et al. 2022).

Li and colleagues demonstrated that vitamin E's antioxidant properties can restore FKBP12.6 levels by shielding the myocardium from oxidative stress. Vitamin E's defensive mechanism against oxidative stress contributes to reducing FKBP12.6 levels in heart failure (Li et al. 2008). In a study conducted on PC12 cells, it was found that pretreatment with vitamin E activated the Nrf2 signaling pathway. By upregulating genes involved in antioxidant defense, vitamin E can help counteract oxidative damage and protect neurons from damage‐related diseases (Huang et al. 2019).

This research aimed to conduct the initial investigation into the impact of diverse dosages of vitamin E augmentation on histopathological alterations, changes in average weight, cognitive behaviors, and the manifestation of age‐associated genes including sirt1, Nrf2, and calstabin2 in the heart and hippocampus of one‐year‐old mice.

Furthermore, we measured oxidative stress indicators, such as SOD, GPX, and MDA, in the heart and brain tissues.

## 2 Materials and Methods

### 2.1 Animals and Treatments

The study involved 32 male NMR mice, approximately 12 months old and weighing 30–40 g, which were kept in the same environmental conditions at the Laboratory Animal Facility of the Rafsanjan University of Medical Sciences (Zhang et al. 2022). Throughout the experiment, the mice were subjected to consistent conditions, including a temperature of 22 ± 2°C, a humidity level of 60%, and a 12‐h light–dark cycle with illumination beginning at 8:00 a.m. They were given unrestricted access to standard pellet chow and water (Zamanian et al. 2017).

Following a one‐week adjustment phase, the mice were arbitrarily allocated to four experimental groups, each consisting of 8 mice:

- Group 1 (control): old mice (12 months) who did not receive Vit E, but in order to equalize the stress of the gavage process compared to other groups, received tap water. By administering oral gavage daily for a period of 4 weeks (Zarei et al. 2024).
- Group 2: Old mice (12 months) were administered a daily oral gavage of vitamin E (Behsa Co., Iran) at a dosage of 100 mg/kg body weight for 28 days.
- Group 3: Old mice (12 months) received a daily oral gavage of vitamin E at a dosage of 200 mg/kg/BW for a duration of 4 weeks.
- Group 4: Old mice (12 months) received a daily oral gavage of vitamin E at a dosage of 400 mg/kg/BW for a duration of 4 weeks (Zarei et al. 2024; Mahdinia et al. 2021).

The Rafsanjan University of Medical Sciences' Animal Ethics Committee sanctioned the study protocol, and the guidelines outlined in The Manual for the Care and Utilization of Laboratory Animals (Institute for Laboratory Animal Research, National Research Council, Washington, DC, National Academy Press, No. 85‐23, updated 1996) were adhered to (Approval ID: IR.RUMS.AEC.1401.003). The study was conducted in accordance with the Basic & Clinical Pharmacology & Toxicology policy for experimental and clinical studies (Tveden‐Nyborg et al. 2023).

### 2.2 Cognitive Function

Following the treatment period, the y‐maze behavioral test was conducted to assess and compare the spatial cognition and short‐term memory capabilities of the animals. The labyrinth used in this test was a Y‐shaped structure made of plexiglass, with three arms measuring 40 × 30 × 15 cm each, connected at an angle of 120°. To conduct the test, each mouse was positioned in the middle of the labyrinth and given the freedom to investigate all areas for 8 min. The number of times the animals entered each arm was recorded using a digital camera. An entry into an arm was considered when the animal's rear legs were completely inside the arm. Correct alternation behavior is characterized as a successful sequential entry into all three arms of the maze, in sets of three. The percentage of alternation was then calculated using the following formula. (Hughes 2004) During the test, the experimenter was placed far away from the Y‐shaped maze so that the smell of the person was not a stimulus for the animals, and before each mouse entered, the maze screen was sanitized with 70% ethyl alcohol.

### 2.3 Harvesting and Sample Preparation

After a 24‐h period following the behavioral tests, the mice underwent anesthesia via intraperitoneal administration of ketamine and xylazine. They were then euthanized by guillotine. The brains and hearts were immediately removed and rinsed with chilled saline solution (0.9%, w/v). One hemisphere of the brain and half of the heart tissue were preserved in 10% formalin for histopathological assessment. The remaining brain and heart tissues were preserved at −80°C for bioanalytical examination. (The dissection of the hippocampus from the brain had been performed prior to these procedures.)

$$\text{Percent Alternation}=\mathrm{PA}=\frac{X}{Y-2}\times 100$$

*X* = The total number of correct alternation behaviors in sets of three; *Y* = The total number of arms that the animal entered.

### 2.4 Cardiac and Brain Histopathological Study

To prepare the tissue samples of the heart and brain for analysis, they were first sectioned to a thickness of 5 μm. These sections were then deparaffinized using 100% xylene, followed by rehydration with ethanol. Serial slices (5 μm in thickness) underwent staining using hematoxylin and eosin (H&E). Histological alterations in the brain and heart were examined with a microscope fitted with an OLYMPUS B 51 camera.

### 2.5 Oxidative Stress Evaluation

The samples were subjected to homogenization in chilled PBS buffer (100 mM, pH 7.4) at a proportion of 1:10 (w/v), followed by centrifugation at 6000 rpm for a duration of 20 min at 4°C to procure the supernatant for the ensuing bioanalytical examination. Levels of MDA, SOD activity, and GPx activity were determined using commercially available kits (ZellBio, Lonsee, Germany) following the manufacturer's guidelines. The measurement ranges for MDA, SOD, and GPx were 0.78–100 μM, 5–100 U/mL, and 20–500 U/mL, respectively. An ELISA Microplate Reader (Rayto, Shenzhen Guangdong, China) was used to record absorbance readings, and each sample was analyzed twice.

### 2.6 Gene Expressions in Hippocampus and Heart by Quantitative RT‐PCR Analysis

To evaluate hippocampal and cardiac tissue mRNA concentrations, total RNA was initially isolated using Parstous Co's RNA extraction kit in Iran. RNA purity and concentration were subsequently ascertained using a Denovix DS‐11 Nanodrop. This RNA underwent conversion to cDNA through the Easy TM cDNA Synthesis Kit, sourced from the same company. Quantification of mRNA expression was conducted using quantitative real‐time PCR (qRT‐PCR) and particular genetic primers as detailed in Table 1. The qRT‐PCR conditions included a primary phase at 95°C for 60 s, followed by 40 cycles at 94°C for 5 s and 60°C for 40 s, concluding with an extension phase at 60°C for 30–20 s. We used the 2 − (ΔΔ*C*_t_) method for mRNA level quantification, with β‐actin as the control gene (refer to Table 1).

**TABLE 1:** Primer sequences.

Gene description	Primer sequence (5′→3′)
SIRT1	Forward: TCTGAAAGTGAGACCAGTAGC
	Reverse: ATGAAGAGGTGTTGGTGGCA
Calstabin2	Forward: TCAGAATTGGCAAACAGGAAGTC
	Reverese: TGAGCAGCTCCACGTCAAAG
Nrf2	Forward: CTCACCTCTGCTGCAAGTAGC
	Reverse: AACTTGTACCGCCTCGTCTG
B‐Actin	Forward: GTACTCTGTGTGGATCGGTG
	Reverse: GGGTGTAAAACGCAGCTCAG

### 2.7 Statistical Analysis

Data analysis was conducted utilizing SPSS software, version 22 (SPSS, Chicago, IL, USA), with outcomes presented as mean ± SEM. Group variances in the experiments were assessed through ANOVA, followed by Tukey's subsequent examination. A *p*‐value less than 0.05 was deemed to suggest statistical importance.

## 3 Results

### 3.1 The Effect of Different Doses of Vitamin E on Working Memory

**FIGURE 1:**
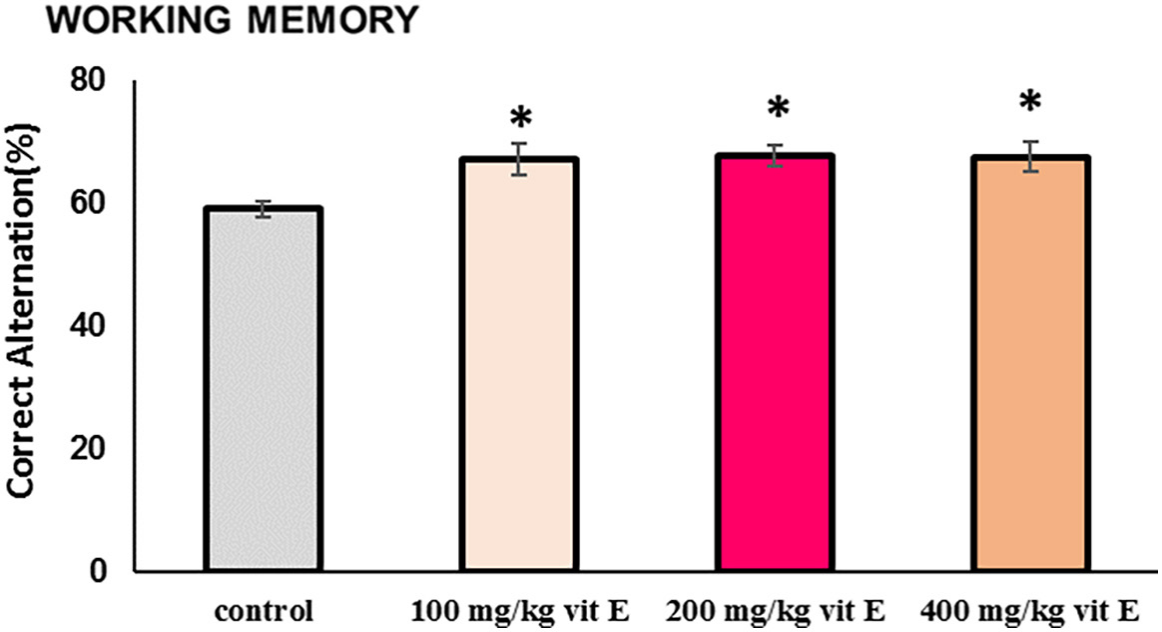
The impact of varying quantities of vitamin E on working memory. Values represent mean ± SEM (*n* = 8 in each group). *p < 0.05 in comparison to control group.

The Y‐maze examination was utilized to evaluate working memory across different test groups. A notable elevation in the percentage of accurate alternations was observed in groups receiving vitamin E, in contrast to the control group, as illustrated in Figure 1. Intriguingly, all three administered doses demonstrated comparable effectiveness, with increased dosages not yielding further improvements in efficacy (*p* < 0.05).

### 3.2 Histopathological Changes in the Heart

**FIGURE 2:**
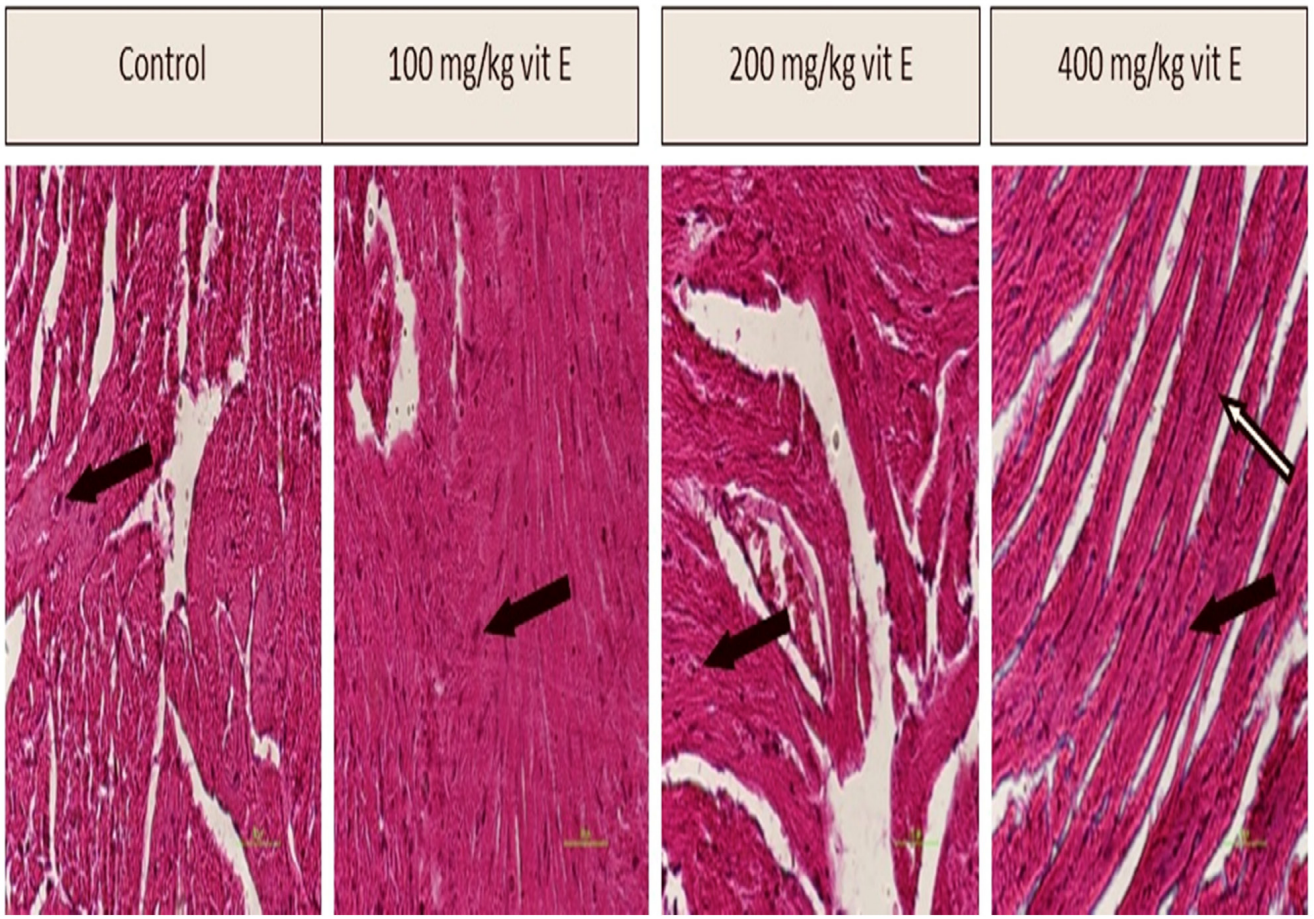
The impact of varying quantities of vitamin E on heart tissue (40×). The black arrows in the picture point to the nucleus of the heart cells and white arrow points to the arrangement of actin and myosin filaments.

H&E staining of cardiac tissue demonstrated no notable differences among the groups administered 100 and 200 mg/kg of vitamin E relative to the control group. However, in the group administered a dosage of 400 mg/kg, the nuclei of the heart cells appeared elongated and euchromatic. Furthermore, the arrangement of actin and myosin filaments, as well as the dark and light bands, in the heart cells of this group exhibited a more regular pattern compared to the other groups (Figure 2).

### 3.3 Histopathological Changes in the Brain

**FIGURE 3:**
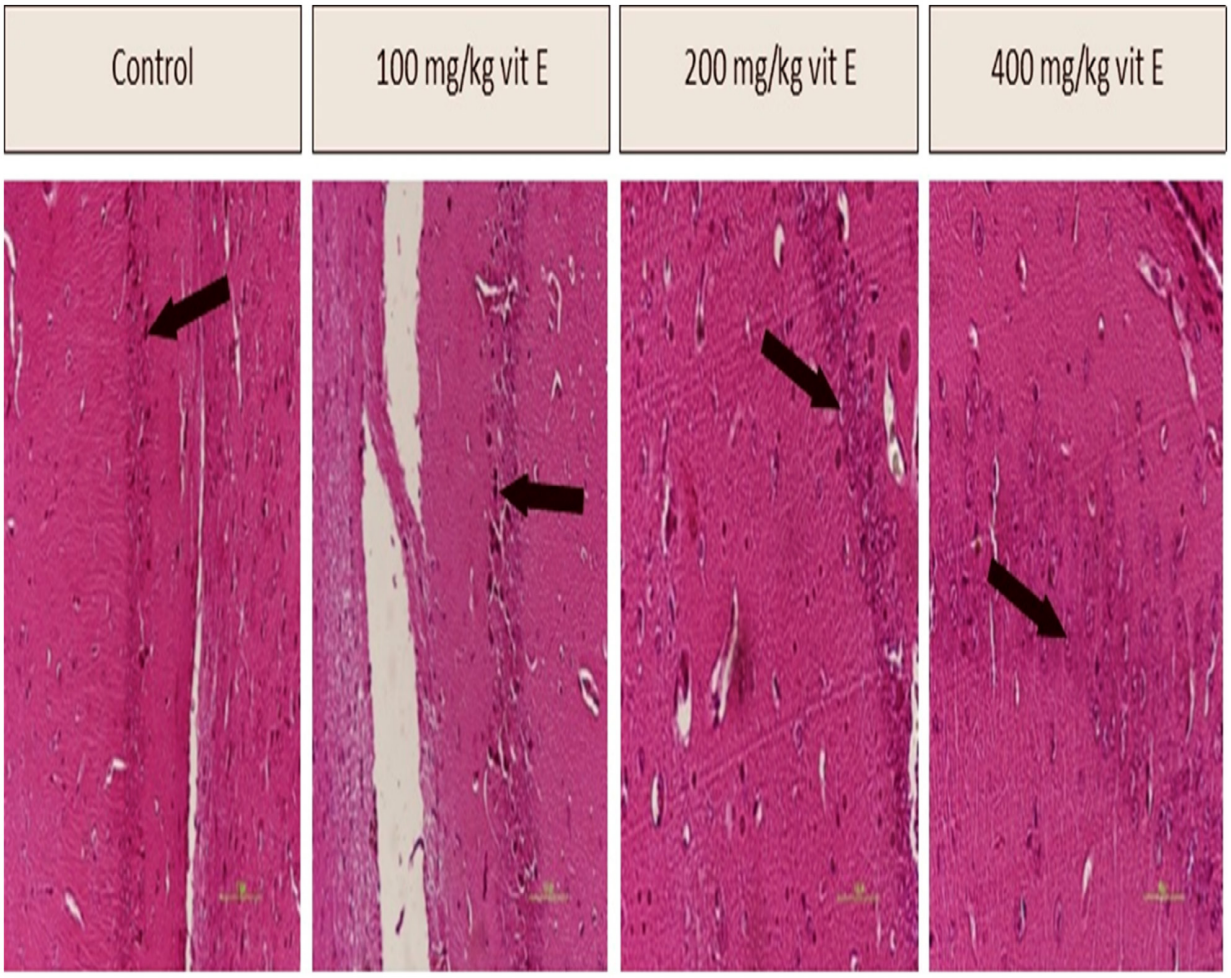
The effect of different doses of vitamin E on brain tissue (40×). The black arrows in the picture point to the euchromatin nuclei the A3 region of the hippocampus.

H&E staining of the brain revealed that in all groups receiving vitamin E, more euchromatin nuclei and bright euchromatin neurons with clear nuclei were observed in the A3 region of the hippocampus in comparison to the control group. Additionally, the effect was particularly noteworthy in the groups administered 200 and 400 mg/kg doses of vitamin E (Figure 3).

### 3.4 The Influence of Diverse Vitamin E Quantities on the Functions of SOD and GPx, in Addition to the MDA Concentrations in the Cerebral and Cardiac Tissues of Elderly Mice

**FIGURE 4:**
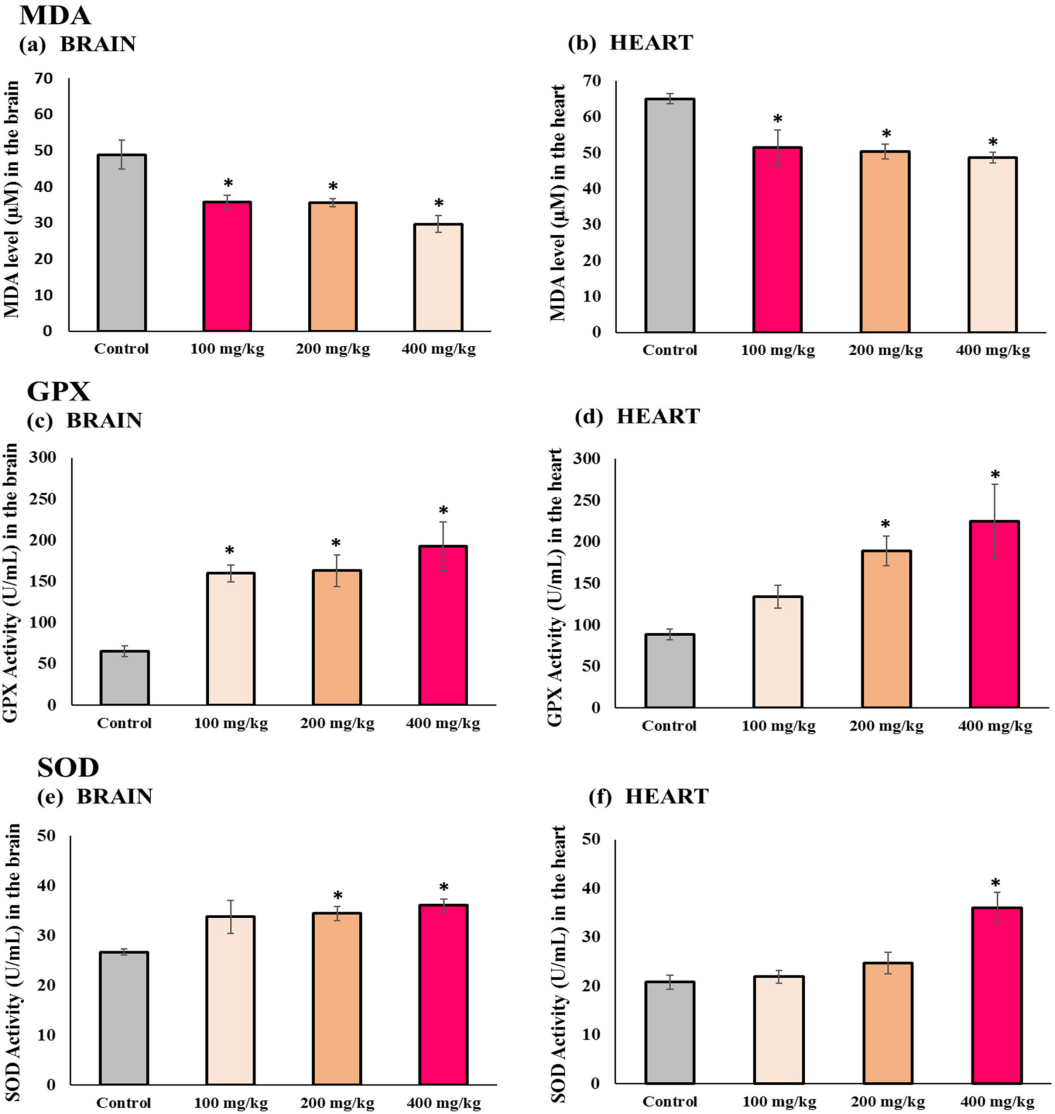
Oxidative stress—MDA levels, and SOD and GPx enzymatic activities. (a) Brain MDA levels; (b) heart MDA levels; (c) brain GPX activity; (d) heart GPX activity; (e) brain SOD activity; and (f) heart SOD activity. Results are presented as mean ± SEM (*n* = 8 in each group). *Significant difference observed at *p* < 0.05 relative to the control group.

To assess heart and brain oxidative stress, we analyzed MDA levels, SOD, and GPX enzyme activities in tissue homogenates. We noted a pronounced, dose‐responsive reduction in MDA concentrations in both heart and brain tissues in all sets treated with vitamin E, compared to the control group (*p* < 0.05). This observation implies that increased doses of vitamin E are more effective in lowering MDA levels (Figure 4a,b).

Analysis of GPX and SOD enzymatic activities in both heart and brain tissues unveiled a substantial elevation across all groups treated with vitamin E compared to the control group (*p* < 0.05). Moreover, this increase was dose‐dependent, indicating that higher vitamin E dosages, especially at 400 mg/kg, markedly boosted the activities of these enzymes (Figure 4c–f).

### 3.5 The Impact of Different Doses of Vitamin E on SIRT1, Nrf2, and calstabin2 Expression in Hippocampus and Heart Tissues of Aging Mice

**FIGURE 5:**
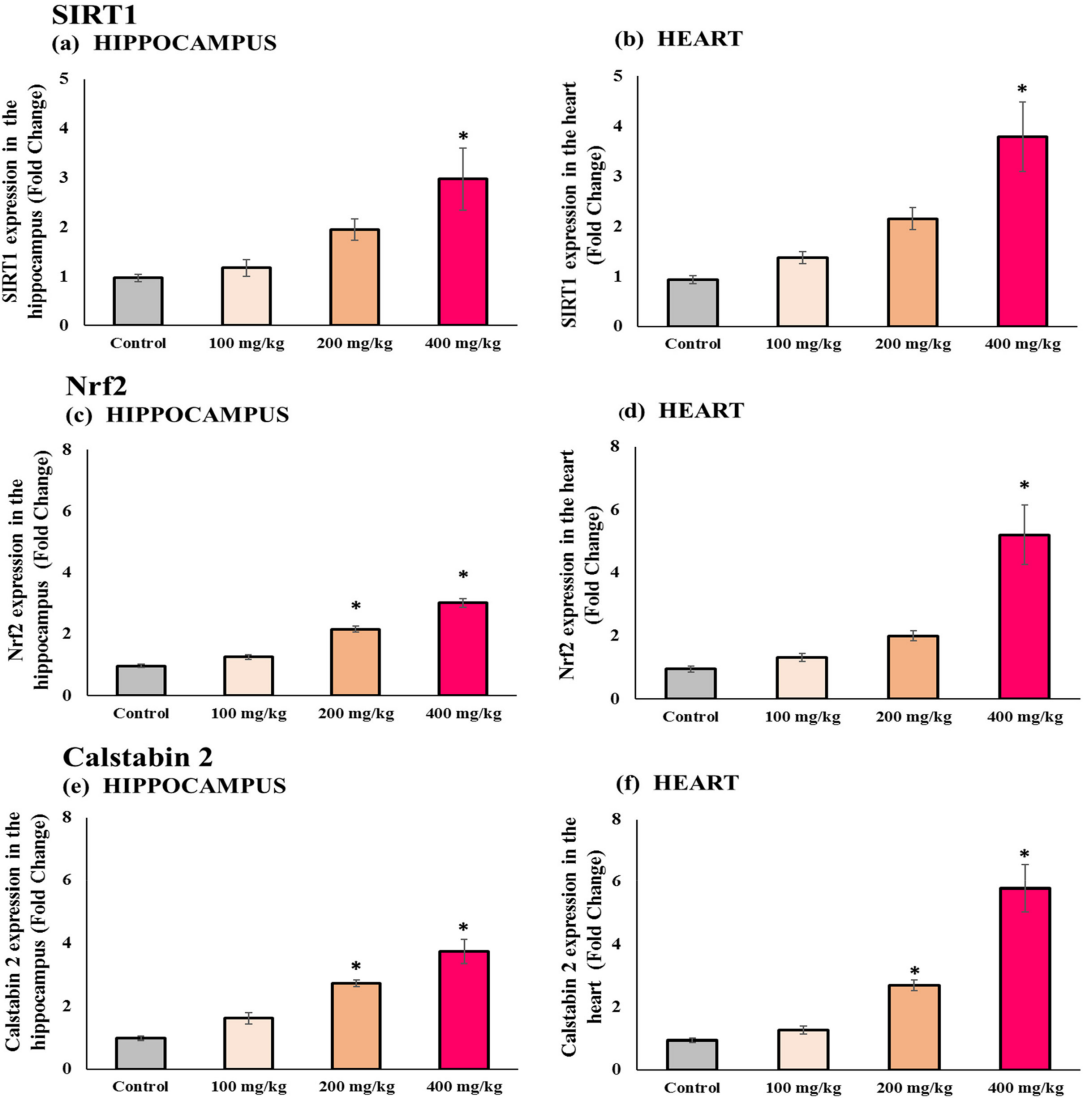
Gene Expression—SIRT1, Nrf2, and Calstabin2 mRNA expression. (a) Hippocampal SIRT1 mRNA expression; (b) cardiac SIRT1 mRNA expression; (c) hippocampal Nrf2 mRNA expression; (d) cardiac Nrf2 mRNA expression; (e) hippocampal Calstabin2 mRNA expression; and (f) cardiac Calstabin2 mRNA expression. Results are presented as mean ± SEM (*n* = 8 in each group). *Significant difference observed at *p* < 0.05 relative to the control group.

The levels of gene expression for SIRT1, Nrf2, and Calstabin2 demonstrated gradual rises depending on the dose in both the hippocampus and heart tissues of the vitamin E‐treated groups relative to the control group. This indicates that higher doses of vitamin E corresponded to a more pronounced enhancement of gene expression. However, the impact of the 100 mg/kg dose of vitamin E did not notably stand out in either tissue. Furthermore, the 200 mg/kg dose showed less efficacy in increasing the expression of the SIRT1 gene in the heart and brain and enhancing the expression of Nrf2 in the heart (Figure 5).

### 3.6 The Effect of Different Doses of Vitamin E on Average Body Weight Changes

**FIGURE 6:**
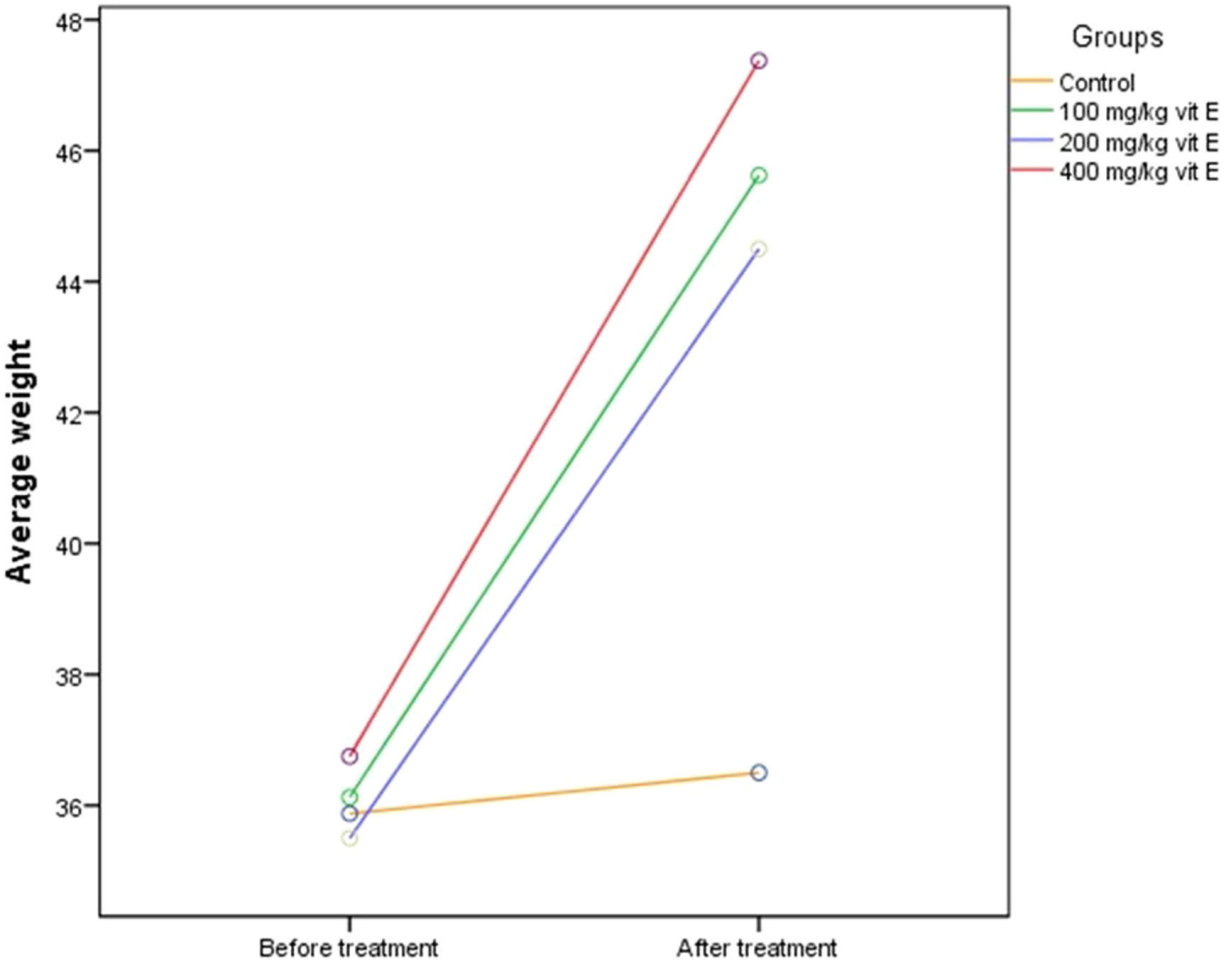
The effect of different doses of vitamin E on average body weight changes before and after treatments. Values are mean *±* SEM (*n* = 8 in each group).

The average weight of mice in each group was compared pre‐ and post‐intervention. The results showed a significant increase in average weight across every group that obtained vitamin E, as compared to the control group. Interestingly, all three doses of vitamin E exhibited nearly equal effectiveness, indicating that increasing the dosage did not lead to a greater weight gain (Figure 6).

## 4 Discussion

The primary objectives of this investigation were to analyze the influence of varied doses of vitamin E supplementation on multiple aging markers. Specifically, the study aimed to evaluate vitamin E's impact on oxidative stress, spatial learning, working memory, and the expression of aging‐related genes such as SIRT1, Nrf2, and calstabin2 in the heart and hippocampus of aged mice. Additionally, the study sought to evaluate the impact of three varied dosages of vitamin E (100, 200, and 400 mg/kg) on age‐related indicators. Additionally, the research aimed to gauge the potential therapeutic advantages of vitamin E supplementation in alleviating age‐related effects on the heart and brain tissues of aged mice.

This research demonstrates that supplementation with vitamin E enhanced working memory and positively impacted the neuronal health of both the hippocampus and cardiac cells. Furthermore, vitamin E supplementation led to a decrease in oxidative stress within the heart and brain, as evidenced by decreased MDA levels, increased SOD and GPX activities, and enhanced expression of SIRT1 and Nrf2 genes. Notably, this study is the first to reveal that vitamin E positively influenced the regulation of calcium homeostasis in both the brain and heart by upregulating the expression of the Calstabin2 gene. The effectiveness of vitamin E was notably influenced by the dosage administered, with higher doses, particularly at 400 mg/kg, demonstrating more beneficial effects.

Aging is the gradual decline and deterioration of physiological functions over time, leading to an elevated susceptibility to cardiovascular and neurodegenerative diseases (da Costa et al. 2016). Oxidative stress is a significant hallmark of aging. Numerous studies have indicated that using antioxidants can exert a beneficial influence on mitigating the detrimental effects of oxidative stress (Salisbury and Bronas 2015; Liguori et al. 2018). Vitamin E not only acts as a cellular protector and aids in preventing degenerative and inflammatory processes, but is also widely recognized for its potent antioxidant properties (Ochi and Takeda 2015).

With advancing age, there is a decline in the link between the cerebral cortex and the hippocampus, resulting in an imbalanced function of the hippocampus. This imbalance and also increased oxidative stress in the brain hamper the ability to learn new information and remember effectively (Leal and Yassa 2015). So aging, specifically within the hippocampus, might lead to disorders in spatial cognition and recall (Juliandi et al. 2021). As shown in the current study, the vitamin E supplementation, as a powerful antioxidative agent, significantly improved both memory and spatial learning in aged mice, as gauged by the correct variation in the Y‐maze. Interestingly, the efficacy of vitamin E was similar across varying doses (100, 200, and 400 mg/kg), indicating that higher dosages did not necessarily yield greater improvements. The results confirm previous similar research, such as research carried out by Baghcheghi et al., which considered the dispensation of vitamin E can mitigate oxidative damage to the brain and ameliorate learning and memory impairments (Baghcheghi et al. 2018).

Aging in the heart leads to complications such as protein structural damage, myocardial contractility disorders, and collagen deposition. These factors contribute to the deterioration of the heart's structure and function, leading to various cardiovascular diseases (Drosatos 2016; Horn et al. 2012). Studies have shown that antioxidant compounds, such as vitamin E, can improve heart tissue conditions during aging (Tyralla and Amann 2003). Vitamin E can serve as a preventive or therapeutic measure for heart disorders (Dianat et al. 2014). Our study indicates that, whereas dosages of 100 and 200 mg/kg of vitamin E did not exhibit a significant impact on the structure of heart tissue, the dose of 400 mg/kg demonstrated notable improvements in cellular condition. Moreover, it led to an increase in the organization of actin‐myosin and the dark and light lines in heart tissue.

Aging in the brain leads to several complications, including reduced cerebral blood flow, dysfunction of mitochondria and nerve calcium regulation, hippocampal shrinkage, narrowing of blood vessels, and increased perivascular space. These factors contribute to the development of neurological diseases (Mattson and Arumugam 2018). Studies indicate that antioxidant compounds, such as vitamins E, can help maintain brain structure during aging. Vitamin E has similarly been identified as a prospective safeguarding agent against long‐term brain damage (Dobrovolny, Smrcka, and Bienertova‐Vasku 2018). Our study indicates that the dosages of 200 and 400 mg/kg of vitamin E resulted in improved neuronal condition, as evidenced by an increased number of euchromatin nuclei and brighter euchromatin neurons with clearer nuclei.

GPX and SOD are antioxidant enzymes, and their activities tend to decline with age. As individuals age, lipid peroxidation caused by free radicals tends to increase. Consequently, the levels of MDA, which constitutes a byproduct of lipid peroxidation in cells, typically rise with age (Ibrahim et al. 2019). Several studies have emphasized the effectiveness of vitamin E as an antioxidant in modulating the function of antioxidative enzymes in specific pathological conditions. Vitamin E appears to possess the ability to effectively counteract oxidative stress by boosting the activity of antioxidant enzymes and reducing levels of MDA. For example, in research carried out by Ibrahim et al., it was discerned that vitamin E successfully alleviated cardiotoxicity induced by anti‐cancer drugs. This was achieved by enhancing the function of GPX and SOD enzymes while reducing the MDA level (Ibrahim et al. 2019). Our study aligns with these findings, shows that vitamin E resulted in a notable increase in the activity of SOD and GPX enzymes and a decrease in MDA levels. These changes were found to be dose‐dependent, indicating that higher doses, particularly a dosage of 400, exhibited a more pronounced effect. From a statistical standpoint, the dosage of 100 mg/kg of vitamin E did not demonstrate sufficient effectiveness in enhancing GPX cardiac action as well as SOD activity in the brain and heart. Furthermore, the dosage of 200 mg/kg of vitamin E did not yield A substantial augmentation in SOD function in the cerebrum, based on statistical analysis.

Multiple studies have indicated reduced levels of SIRT1 during the aging process (Kwon et al. 2017; Chen et al. 2020; Yuan et al. 2016a). SIRT1, a distinct sirtuin variant, functions as a deacetylase and holds significant importance in retarding cellular aging and prolonging lifespan by deacetylating specific vital substrates (Pang et al. 2019; Kou et al. 2016). Generally, sustaining SIRT1 levels provides protection to endothelial cells, the heart (Hsu et al. 2008), and the brain, as well as against age‐related diseases (Khan et al. 2019). Multiple research investigations have suggested a potential positive influence of vitamin E on elevating sirt1 expression (Rad et al. 2019; Saboori et al. 2016). Our study corroborates these observations by revealing that vitamin E intake resulted in a dose‐related elevation of sirt1 gene expression in both brain and heart tissues in comparison to the control group. Nevertheless, only the 400 mg/kg dosage displayed statistically notable alterations in both brain and heart tissues, while the effects seen from doses of 100 and 200 mg/kg were not as prominent.

Nrf2, an essential transcription factor, serves as the key regulator of cellular redox homeostasis. It plays a crucial role in neutralizing harmful free radicals and their detrimental effects by regulating its target genes. Nonetheless, it's crucial to acknowledge that the manifestation of this gene tends to decline with aging (Schmidlin et al. 2019). Similar studies in this field predominantly highlight the positive impact of vitamin E on the expression of Nrf2. For instance, Fang et al. showed that a 100 mg/kg dose of vitamin E resulted in improved liver oxidative stress and increased Nrf2 expression in rats with cadmium chloride toxicity and severe oxidative stress in the live (Fang et al. 2021). Our study aligns with these findings, indicating that vitamin E could lead to increased Nrf2 gene expression in both tissues. However, only the dosage of 200 and 400 mg/kg of vitamin E in the hippocampus and the dosage of 400 mg/kg of vitamin E in the heart showed statistically significant changes, and the effects observed from other doses were not as significant.

A notable transformation associated with aging involves modifications in the calcium signaling pathway (Santulli and Marks 2015). Present in both the hippocampus and heart is the calcium discharge pathway known as Ryanodine Receptor 2 (RYR2), which plays a crucial role in regulating contractions (Yuan et al. 2016b; Fill and Copello 2002). Calstabin2 is a regulatory protein that binds to the RYR2 receptor complex, stabilizing its closed form. This binding prevents the improper leakage of calcium, ensuring proper nerve and muscle function. Moreover, Calstabin2 also contributes to the regulation of the learning and memory processes (Yuan et al. 2014). Due to aging‐induced oxidative stress, the expression of the calstabin2 gene diminishes. A limited number of research works have investigated the effects of vitamin E on calstabin2 expression. Our research, notably the first of its kind, reveals the potential of vitamin E to positively influence calcium regulation in the brain and heart by augmenting calstabin2 gene expression. However, further investigations are imperative to validate these outcomes. Our findings indicated a dose‐dependent elevation in calstabin2 gene expression in both brain and heart tissues. Notably, only doses of 200 and 400 mg/kg exhibited considerable significant changes, whereas the effects observed with the 100 mg/kg dosage of vitamin E were less significant.

As evidenced by the current investigation, there was a notable increase in average weight observed in the vitamin E‐treated groups compared to the control group following the therapy. Nevertheless, we did not detect any substantial variations in the impact of different doses on weight gain. All three doses were nearly equally effective in promoting weight gain. The results confirm previous similar research, such as a study conducted by Haung et al. on a particular fish species, which demonstrated that supplementing with vitamin E resulted in elevated weight gain and feed consumption among the fish (Huang et al. 2020).

## 5 Conclusions

The present study showed that vitamin E supplementation can yield notable positive effects, even during advanced age. This vitamin can improve working memory and spatial learning, as indicated by increased accuracy in maze test performance. Moreover, vitamin E can diminish oxidative harm in the brain and heart tissues and slow down the aging process by augmenting the function of antioxidant catalysts, decreasing lipid peroxidation, and promoting the expression of sirt1 and Nrf2 genes. This vitamin may also have a positive effect on regulating calcium homeostasis in the brain and heart by increasing the expression of the calstabin2 gene. However, further studies are necessary to confirm these findings in this particular field. On the other hand, vitamin E has been found to have positive effects on neuronal health in the hippocampus and heart cells. The efficacy of vitamin E is mainly directly influenced by the dosage administered. Higher doses, particularly at 400 mg/kg, are associated with more favorable effects.

## Author Contributions

**Farnoosh Molavi Vasei:** data curation (equal), methodology (equal), software (equal), writing – original draft (equal). **Mohammad Yasin Zamanian:** writing – review and editing (equal). **Maryam Golmohammadi:** writing – original draft (equal). **Mehdi Mahmoodi:** visualization (equal), writing – original draft (equal). **Morteza Khademalhosseini:** methodology (equal), resources (equal). **Tayyebeh Tavakoli:** formal analysis (equal), methodology (equal). **Ozra Sadat Esmaeili:** data curation (equal), methodology (equal), software (equal). **Sadegh Zarei:** methodology (equal). **Mohammad Reza Mirzaei:** writing – review and editing (equal). **Mohammad Reza Hajizadeh:** conceptualization (equal), investigation (equal), project administration (equal), supervision (equal), validation (equal), visualization (equal), writing – review and editing (equal).

## Conflicts of Interest

The authors declare no conflicts of interest.

## Data Availability

Data underpinning this study's conclusions is available upon request from the corresponding author.
